# Adenylate Kinase 3 Sensitizes Cells to Cigarette Smoke Condensate Vapor Induced Cisplatin Resistance

**DOI:** 10.1371/journal.pone.0020806

**Published:** 2011-06-15

**Authors:** Xiaofei Chang, Rajani Ravi, Vui Pham, Atul Bedi, Aditi Chatterjee, David Sidransky

**Affiliations:** Department of Otolaryngology-Head and Neck Surgery, Johns Hopkins University School of Medicine, Baltimore, Maryland, United States of America; University of Illinois at Chicago, United States of America

## Abstract

**Background:**

The major established etiologic risk factor for bladder cancer is cigarette smoking and one of the major antineoplastic agents used for the treatment of advanced bladder cancer is cisplatin. A number of reports have suggested that cancer patients who smoke while receiving treatment have lower rates of response and decreased efficacy of cancer therapies.

**Methodology/Principal Findings:**

In this study, we investigated the effect of cigarette smoke condensate (CSC) vapor on cisplatin toxicity in urothelial cell lines SV-HUC-1 and SCaBER cells. We showed that chronic exposure to CSC vapor induced cisplatin resistance in both cell lines. In addition, we found that the expression of mitochondrial-resident protein adenylate kinase-3 (AK3) is decreased by CSC vapor. We further observed that chronic CSC vapor-exposed cells displayed decreased cellular sensitivity to cisplatin, decreased mitochondrial membrane potential (ΔΨm) and increased basal cellular ROS levels compared to unexposed cells. Re-expression of AK3 in CSC vapor-exposed cells restored cellular sensitivity to cisplatin. Finally, CSC vapor increased the growth of the tumors and also curtail the response of tumor cells to cisplatin chemotherapy *in vivo*.

**Conclusions/Significance:**

The current study provides evidence that chronic CSC vapor exposure affects AK3 expression and renders the cells resistant to cisplatin.

## Introduction

As per NCI records in United States alone, there are an estimated 70,980 new cases and 14,330 bladder cancer related deaths each year, making it the fourth most common cancer in men and eighth most common cancer in women. Cigarette smoking has been recognized as the main cause of bladder cancer and accounts for almost 50% of the cases in developed countries [1]. Over 4000 compounds have been identified in cigarette smoke, 60 of which have been found to be complete carcinogens, tumor initiators, promoters and/or co-carcinogens in various in vitro and animal bioassays [2]. Secondhand smoke, also known as passive smoke or environmental tobacco smoke, has been also associated with bladder cancer, and is the cause of about 38,000 deaths annually in America alone [3]. Reports by the International Agency for Research on Cancer (IACR) have classified secondhand tobacco smoke (passive smoking) as an established human carcinogen [4]. There are over 4000 chemical compounds in secondhand smoke; 200 of which are known to be poisonous, and upwards of 60 have been identified as carcinogens [5], [6]. Exposure to secondhand smoke or even just a past smoking history can also increase the likelihood of bladder cancer in offspring [7].

One of the most commonly used antineoplastic agents for the treatment of advanced bladder cancer is cisplatin, but the development of resistance to cisplatin during treatment is common and constitutes a major obstacle to the cure of sensitive tumors [8]. Although many studies have been conducted on the molecular mechanism of drug resistance, little is known about the treatment of these drug-resistant tumors, which still remains a significant problem. A number of reports have suggested that cancer patients who smoke while receiving treatment have poorer outcomes compared with their nonsmoking counterparts, possibly because of lower rates of response [9]. Retrospective series of patients with renal[10], bladder[11], and especially glottic cancers[12], [13] also indicate a link between smoking during treatment and decreased efficacy of cancer therapies. However, there are no direct data showing that cigarette smoke could actually induce resistance to chemotherapeutic agents, such as cisplatin.

Deletion of chromosome 9p frequently occurs in bladder tumors. Depending on the respective investigation, frequencies between 30 and 70% have been published [14], [15]. Studies by Blaveri *et al*., [16] indicate that in primary bladder cancer, loss of clones across the entire chromosome 9 occurs with an average frequency of 47% for 9p and 46% for 9q. Chromosome 9 carries important genes involved in adenine metabolism, namely AK1, AK2 and AK3 (adenylate kinase 1,2 and 3). All three AKs are nuclear-encoded proteins and synthesized in the cytoplasm. AK1 remains located mainly in the cytosol of different tissues. Mature AK2 and AK3 are imported into mitochondria, where AK2 resides in the intermembrane space whereas AK3 is located exclusively in the mitochondrial matrix [17]. Cigarette smoke is also known to induce mitochondrial damage as well as dysfunction [18], [19] which may in turn increase cisplatin resistance in bladder cancer cells [20]. In this study we examined the relationship between tobacco exposure and cisplatin resistance in relation to mitochondria function and specifically to a mitochondria-resident protein AK3. We showed here that cigarette smoke condensate (CSC) vapor can induce cisplatin resistance. We also found that the expression of AK3 is affected by CSC vapor and that restoration of this gene sensitizes the cells to cisplatin.

## Results

### CSC vapor decreased the expression of AK3 and increased cisplatin resistance

**Figure 1:**
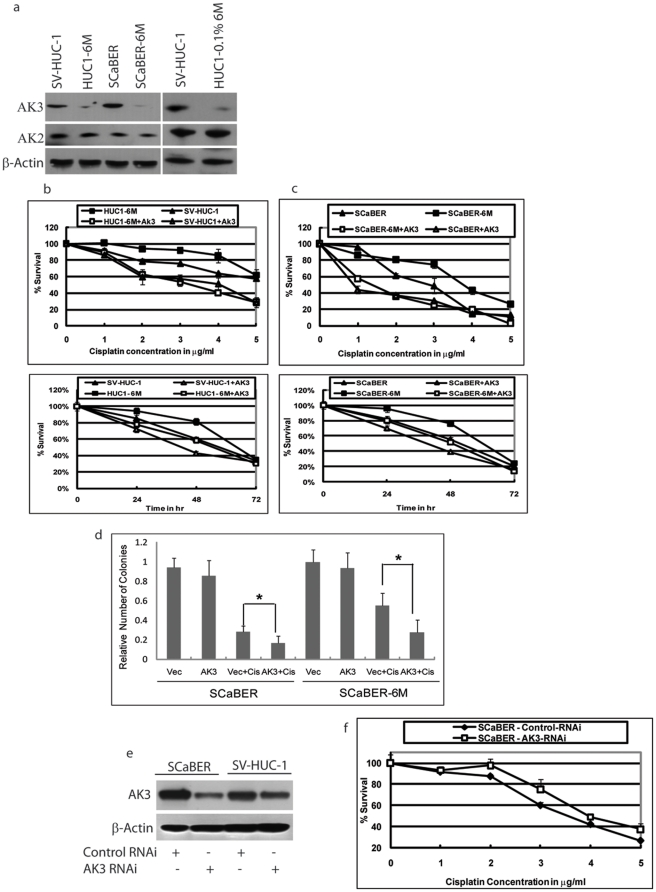
CSC vapor decreased the expression of AK3 and increased cisplatin resistance. (a) Western blot analysis was performed using anti-*AK3* and anti-*AK2* antiserum. Protein extracts in each lane are as indicated. Even loading was confirmed by re-probing the membrane with β-Actin antibody. HUC1-6M or SCaBER-6M indicates the cells chronically exposed to CSC vapor. HUC1-0.1%6M are SV-HUC-1 cells exposed to 0.1% CSC for 6 months. SV-HUC-1 and HUC1-6M (b), SCaBER and SCaBER-6M (c) were treated with 0 to 5 µg/ml cisplatin for 48 h, or with 3 µg/ml cisplatin for 24, 48 and 72 h, in the presence and absence of AK3 as indicated. (d) Colony formation assays were performed with SCaBER and SCaBER-6M in the presence and absence of AK3 as indicated. Data were expressed as mean ±SD. * indicates *P*<0.05 (e) SV-HUC-1 and SCaBER cells were transfected with AK3 RNAi for 48 h and Western blot analysis was performed using anti-AK3 antiserum. (f) SCaBER cells were transfected with AK3 RNAi and 48 h after transfection the cells were treated with 0 to 5 µg/ml cisplatin for 48 h and cellular survival was assessed. Error bars represent standard deviation of three experiments.

Human urothelial immortal cell line SV-HUC-1 and bladder cancer cell line SCaBER was exposed to CSC vapor (passive smoke) for 6 months and were then designated as HUC1-6M and SCaBER-6M. We investigated cisplatin sensitivity and mitochondrial-residing genes AK2 and AK3 in response to CSC vapor exposure. In both cell lines, we found that CSC vapor decreased AK3 expression, whereas AK2 was not affected (Figure 1a). We also noted that treatment of SV-HUC-1 cells with 0.1% of CSC for 6 months (HUC1-0.1%6M) also led to a decrease in expression of AK3 but not AK2. We examined the effect of cisplatin on SCaBER and SV-HUC-1 cells. Chronic exposure of the cells to CSC vapor had made the SV-HUC-1 and SCaBER cells resistance to cisplatin (Figure 1b and 1c). To study the effect of AK3 expression on the cellular survival of bladder cells in response to cisplatin, we cloned the full length of AK3 and transfected the construct into SV-HUC-1, HUC1-6M, SCaBER, and SCaBER-6M cells. 24 h after transfection with AK3 expression vector or the empty control vector, cells were treated with or without cisplatin and survival was assessed by the MTT assay. Treatment of SV-HUC-1 cells with cisplatin revealed that the cells had acquired resistance to cisplatin upon exposure to CSC vapor (Figure 1b, compare the solid square to solid triangle). Over-expression of AK3 in the HUC1-6M resulted in a significant increase in the sensitivity of the cells to cisplatin (Figure 1b, compare solid square to open square). Akin to the results of SV-HUC-1, the SCaBER-6M cells too had acquired resistance to cisplatin compared to SCaBER parental cells (Figure 1c, compare solid square to solid triangle). Over-expression of AK3 in the SCaBER-6M cells resulted in a significant amount of cell death in response to cisplatin, rendering the cells more sensitive to cisplatin (Figure 1c, compare closed square to open square). We also confirmed the survival data with Calcein AM assay due to the fact that MTT results might be subjected to altered mitochondrial function (data not shown). In addition, colony formation assay was performed on SCaBER and SCaBER-6M which showed that SCaBER-6M was more resistant to cisplatin which could be reversed by AK3 (Figure 1d). We next attempted to knock down AK3 in SV-HUC-1 and SCaBER cell lines using AK3 specific RNAi. Western Blot analysis of the cells 48 h after transfection with AK3 RNAi revealed a successful knockdown of AK3 in the SCaBER cells but not in the SV-HUC-1 cells (Figure 1e). Treatment of the cisplatin to these cells revealed that SCaBER cells, but not SV-HUC-1, became resistant to cisplatin in the presence of AK3 RNAi (Figure 1f and data not shown). This is possibly due to the fact that AK3 RNAi transfection failed to produce significant knockdown of the protein in SV-HUC-1 cells. Take together, these data suggest that CSC vapor renders bladder cells resistant to cisplatin and that AK3 expression re-sensitizes these cells to the drug.

To investigate whether the cisplatin sensitivity in these cells further affected after prolonged CSC treatment, we chronically exposed the cells to CSC vapor for 11 months. We observed no significant difference between the cells exposed to CSC vapor for either 6 and/or 11 months (data not shown), and we thus confined the remaining experiments to 6 months treatment only, unless otherwise mentioned.

### ΔΨm Alteration by AK3 in the Presence of Cisplatin

**Figure 2:**
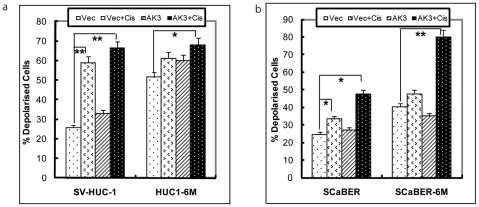
ΔΨm Alteration by AK3 in the Presence of Cisplatin. Mitochondrial membrane potential was measured in SV-HUC1, HUC1-6M (a), SCaBER and SCaBER-6M (b) cells transfected with AK3 or control vector, followed by treatment with cisplatin for 48 h. Cells were stained with JC-1 reagent and cell fluorescence was measured on a flow cytometer using Fl1 and Fl2 channels. Increase of red fluorescence indicates hyper-polarization of mitochondrial membrane potential (ÄΨm). * indicates *P*<0.05 and ** indicates *P*<0.01.

Since AK3 is a mitochondrial-resident protein and also affected cisplatin sensitivity, we next examined how expression of AK3 in combination with cisplatin affects the mitochondrial membrane potential of the bladder cells before or after exposure to CSC vapor. The change in mitochondrial membrane potential (ΔΨm) was monitored by measuring the shift in the fluorescence of the emitted light of JC-1. SV-HUC-1 and HUC1-6M cells were transfected with control vector or AK3 expression plasmid and then treated with vehicle or cisplatin, before JC-1 staining. We found that exposure of SV-HUC-1 cells to CSC vapor for 6 months led to a significant decrease in membrane potential of the cells, consistent with previously reported data [21]. Treatment with cisplatin for 48 hrs resulted in an increase in number of depolarized cells in SV-HUC-1 cells from 25.9% to 58.96%, but to a much lesser extent in HUC1-6M cells. However, when HUC1-6M cells were treated with cisplatin in presence of forced expression of AK3, the number of depolarized cells increased from 51.50% to 68.09% (Figure 2a). Similarly, SCaBER-6M cells were depolarized compared to unexposed parental SCaBER. The SCaBER-6M showed increased depolarization when treated with cisplatin in the presence of AK3 overexpression, but to a much lesser degree with cisplatin alone (Figure 2b).

### ROS Production by AK3 in the Presence of Cisplatin

**Figure 3:**
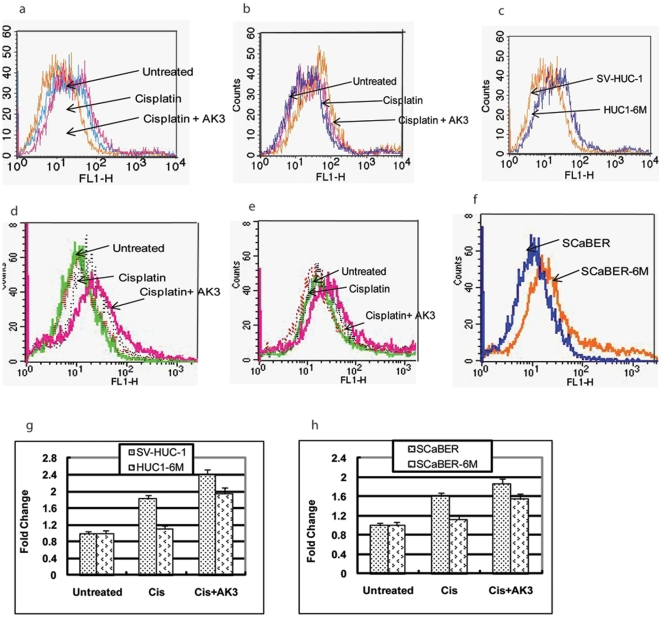
ROS Production by AK3 in the Presence of Cisplatin. ROS production was measured in parental SV-HUC-1 and SCaBER cells or cells exposed to CSC vapor for 6 months using DCFH-DA staining. The cells were transfected with or without the AK3 expression plasmid and treated with or without cisplatin for 48 h before staining. ROS generation in SV-HUC-1 and HUC1-6M (a–c) and SCaBER and SCaBER-6M (d–f) cells with overexpression of AK3 with and without cisplatin. The mean fluorescence density was calculated and data was plotted as mean ± SD (g,h).

We studied the generation of ROS in the parental SV-HUC-1 and SCaBER cells after exposure to cisplatin in the presence or absence of AK3 overexpression. Exposure of the cells to cisplatin alone led to a significant increase in ROS production in the parental SC-HUC-1 and SCaBER cells, which was further enhanced when the cells were treated with cisplatin in the presence of AK3 overexpression (Figure 3a, d). The basal ROS levels in CSC vapor exposed cells HUC1-6M and SCaBER-6M were higher than those in parental cells (Figure 3c, f). As previously noted, HUC1-6M and SCaBER-6M cells had acquired resistance to cisplatin; treatment with cisplatin alone did not increase in ROS production significantly, but treatment of the HUC1-6M and SCaBER-6M cells with cisplatin in the presence of AK3 expression, enhanced the ROS production (Figure 3 b, e). The mean fluorescence density of the cells is depicted in figures 3g and 3 h.

### AK3 Induced Release of Lactate Dehyderogenase upon Treatment of Cisplatin

**Figure 4:**
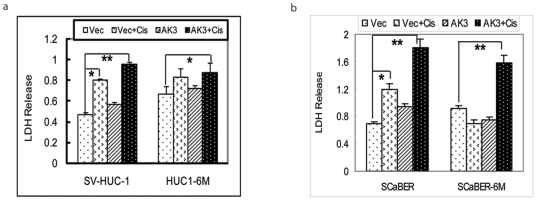
AK3 Induced Release of Lactate Dehyderogenase upon Treatment of Cisplatin. LDH release in SV-HUC-1 (a) and SCaBER (b) cells exposed to CSC vapor in presence of AK3 followed by cisplatin treatment for 48 h. The data are presented as means ± SD. All experiments were performed in triplicate. Statistical significance is as indicated with * indicates *P*<0.05 and ** indicates *P*<0.01

SV-HUC-1, HUC1-6M, SCaBER, and SCaBER-6M cells were transfected with AK3 expression plasmid or the control plasmid. 24 h after transfection, cells were treated with cisplatin and the cytotoxic effect of the drug on the cells was studied by measuring LDH release in the culture medium, as an indicator of cell membrane damage. Treatment of the parental SV-HUC-1 and SCaBER cells with cisplatin alone or cisplatin in presence of AK3 expression plasmid led to significant release of LDH in the media (Figure 4a, b). Exposure to CSC vapor, however, increased the basal levels of LDH in the medium both in SV-HUC1-6M and in SCaBER-6M, compared with parental cell lines. As the CSC vapor exposed cells had acquired resistance to cisplatin, treatment of the HUC1-6M and SCaBER-6M cells with cisplatin alone did not produce any significant change in the release of LDH. When the cells were treated with cisplatin under AK3 overexpression, LDH release was significantly increased in both HUC1-6M and SCaBER-6M cells, indicating increased cellular membrane injury.

### Activation of Apoptosis in the Presence of AK3 and Cisplatin

**Figure 5:**
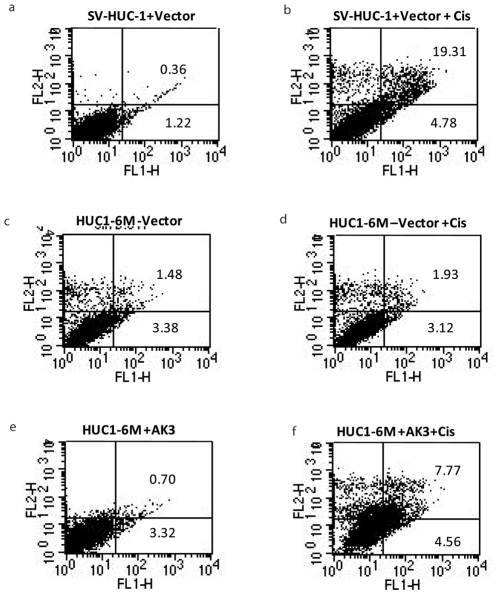
Activation of Apoptosis in the Presence of AK3 and Cisplatin. Apoptosis was measured in the SV-HUC-1 and HUC1-6M cells using annexin/PI staining. The cells were transfected with or without AK3 expression plasmid and treated with or without cisplatin for 48 h as indicated.

Next we studied the induction of apoptosis by cisplatin in SV-HUC-1 and HUC1-6M in the presence or absence of AK3. Apoptosis was determined by staining cells with annexin V-fluorescein isothiocyanate and propidium iodide (PI). 48 hrs of cisplatin treatment induced 19.31% late apoptosis ( both annexin V and PI positive) in SV-HUC-1 cells, with 4.8% of cells undergoing early apoptosis (annexin V positive and PI negative), compared to 0.36% late apoptotic cells and 1.2% of early apoptotic cells in the absence of cisplatin (Figure 5a, b). The same dose of cisplatin did not affect the apoptosis in HUC1-6M cells (Figure 5c, d), consistent with the resistance phenotype of these cells to cisplatin. Overexpression of AK3 alone did not induce any apoptosis (Figure 5e), but importantly, the presence of AK3 potentiated the toxicity of cisplatin by increasing apoptosis (Figure 5f).

### CSC vapor induces Survival Proteins

**Figure 6:**
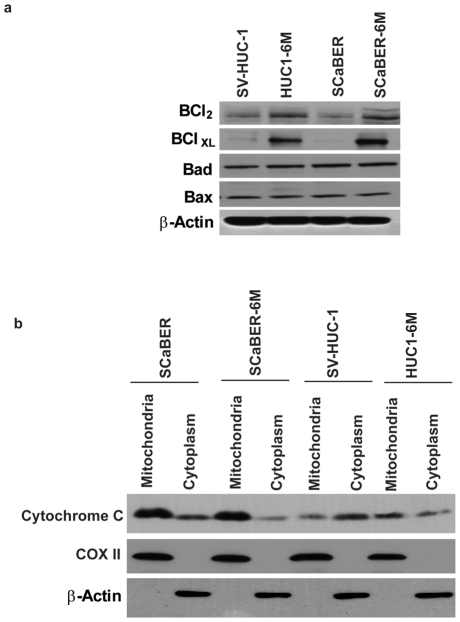
CSC vapor induces Survival Proteins. (a) Cellular lysates were obtained from the indicated cell lines and subjected to Western Blot analysis using the indicated antibodies. β-actin was used as a control. (b) The indicated cell lines were treated with 3 µg/ml cisplatin for 48 h. Mitochondrial and cytoplasmic total cellular lysates were subjected to Western Blot analysis using anti-cytochrome c antibody. Protein extracts in each lane are as indicated. Immunodetection of â-actin and COX II were done to assure that the transfected proteins were in cytoplasm and mitochondria respectively.

As the CSC vapor exposed cells showed activation of pro-apoptotic factors and yet were evading cell death due to apoptosis, we next examined the expression of Bcl-2 family proteins in response to CSC vapor. Western Blot analysis revealed that both Bcl-xL and Bcl-2 protein expression increased in HUC1-6M and SCaBER-6M cells compared to the parental cells (Figure 6a) but the levels of Bax and Bad remained unaffected. Further, we studied the release of cytochrome c in CSC vapor exposed cells in response to cisplatin. Our data showed that there was less cytochrome c release in response to cisplatin both in SCaBER-6M and HUC1-6M cells compared to the parental cells (Figure 6b).

### CSC Vapor Induces Cisplatin Resistance In Xenograft Model

**Figure 7:**
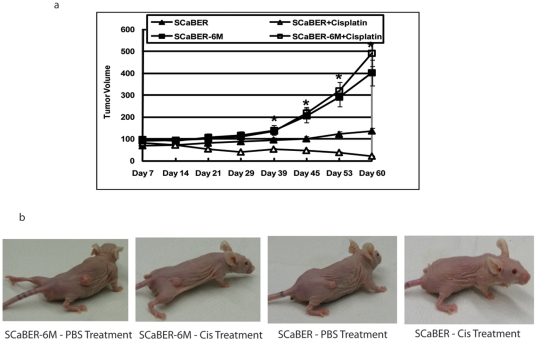
CSC Vapor Induces Cisplatin Resistance In Xenograft Model. (a) Subcutaneous xenografts were generated in of 5- to 6-week-old female athymic *nu/nu* mice with SCaBER or SCaBER-6M cells. One week following tumor cell inoculation, mice were treated with either PBS or cisplatin (3 mg/Kg/dose). The mean tumor volume was calculated. * indicates *P*<0.01 (b) One representative mouse from each group was displayed.

We next tested whether CSC vapor cells are resistance to cisplatin *in vivo*. Athymic nude mice were injected subcutaneously (s.c.) with either parental SCaBER cells or their counterparts SCaBER-6M cells. At day 7 when the tumors reached the size of approximately 50 mm^3^, mice were randomized into two groups of eight animals each and treated with either PBS or cisplatin intraperitoneally. Tumor volumes were measured weekly and the mean tumor volume was calculated. We observed a significant increased growth rate of SCaBER-6M xenografts than the parental ones over the 60-day experimental period (*P*<0.01; Figure 7a). In addition, treatment of mice bearing SCaBER xenografts with cisplatin resulted in a marked inhibition of tumor growth compared to their PBS controls (*P*<0.05, Figure 7a). However, treatment with cisplatin was ineffective in inhibiting the growth of tumors derived from SCaBER-6M cells (Figure 7a).

## Discussion

Secondhand smoke exposure or exposure to environmental smoke has been assessed as a risk of bladder cancer [22]. Our approach here is based on recent studies on normal oral keratinocytes indicating that exposure of cells to vapor component of cigarette smoke extract is as effective as direct treatment of cigarette smoke extract[21]. These studies also indicated that chronic exposure to cigarette smoke provide a better model *in vivo* than acute exposure to cigarette smoke [21]. Studies with environmental cigarette smoke or secondhand smoke in mice have demonstrated a variety of early alterations, including cytogenetic damage in bone marrow and peripheral blood, formation of lipid peroxidation products in lung, increase of bulky DNA adducts and oxidatively generated DNA damage[23], [24].

Our observation in this model that AK3 protein expression is decreased by CSC vapor builds on an old observation that cigarette smoke poisons lung cilia through a direct effect on adenylate kinases [25]. Also increase of *AK3* mRNA level and AK3 enzyme activity were previously observed in rat skeletal muscle [26]. In addition, AK3 protein was found to increase 10-fold during neural differentiation of P19 embryonal carcinoma cells [27]. The induction of *AK3* mRNA was also shown in response to hypoxia in HeLa cells depending on the presence of hypoxia-inducible factor-1 [28]. Here we show a direct effect on cancer cells and provide novel evidence that decreased expression of AK3 in the presence of CSC vapor is accompanied with decreased sensitivity of bladder cells to cisplatin and restoration of AK3 sensitizes cells to cisplatin. By linking AK3, our data support the notion that mitochondria plays an important role in cigarette smoke induced cisplatin resistance. Studies indicate that components present in cigarette smoke extract are able to pass through the membranes of mitochondria[29], [30]. Further it has been proven that highly reactive components like polycyclic aromatic hydrocarbons, aldehydes, phenols, heavy metals, and amines are lipophilic candidates that easily enter the cell and disturb mitochondria [31], [32]. AK3 is present in the mitochondrial matrix and probably functions in transferring the high-energy phosphate to AMP from GTP that is synthesized by the TCA cycle [33], [34]. Decrease of cellular ATP and rapid depolarization of mitochondrial membrane potential and induction of apoptosis by cigarette smoke have been well established in various forms of cancer [30], [35].

The role of mitochondria in cisplatin resistance is further supported by recent data which showed that cisplatin may directly interact with mitochondria [36]. Cisplatin introduces DNA damage by forming inter- and intra-strand nuclear DNA crosslinks. However, only a low percentage of intracellular platinum is bound to nuclear DNA, while a great majority of the intracellular drug interacts with nucleophilic sites on other molecules, including mitochondrial DNA [37], [38]. Mitochondrial DNA-cisplatin adducts may be more common than cisplatin adducts with nuclear DNA in the same cell line treated with the same concentration of cisplatin [39], [40]. Furthermore, Yang *et al* [41] showed that glutathione counteracted the cytotoxicity of cisplatin by preventing ROS production rather than inhibiting formation of platinum/DNA adducts.

Mitochondria are the predominant source of ROS produced in most apoptotic systems and mitochondrial homeostasis is critical in regulating apoptosis [42]. In a normal system, apoptosis at the mitochondrial level is initiated by depolarization of the mitochondrial membrane, followed by release of cytochrome c. Other mitochondrion-related cellular alterations that are important in modulating apoptotic include (but not limited to) induction of ROS. We observed that CSC vapor-selected acquisition of resistance to cisplatin was marked by the presence of decreased mitochondrial membrane potential (ΔΨm, i.e increased depolarization) and increased basal levels of ROS. Comparable levels of ROS generation and ΔΨm depolarization might have resulted in cellular apoptosis and cell death in unexposed cells. However, we also observed that chronic CSC exposed cells are resistant to ΔΨm depolarization-induced apoptosis (data not shown). Similar findings were also reported in an oral keratinocytes [21]. AK3 is a mitochondrial-matrix protein which is downregulated by chronic CSC exposure. We observed decreased ΔΨm (i.e increased depolarization) and increase in ROS production in AK3 overexpressed, cisplatin treated cells. Studies have indicated activation of NF-κB, histone modification and chromatin remodeling in response to CSC [43]. Studies by the same group also indicate an increase in NF-κB in response to ROS [44].

Mitochondrial death signaling via multi domain Bcl-2 family members Bax and Bak can be interrupted by Bcl-xL, an anti-apoptotic member of the Bcl-2 family, which sequesters and counteracts the proaopototic signals [45]. Our results indicate an increase in the expression of Bcl-xL in the CSC vapor exposed cells compared to the parental cells. Bcl-xL has been found to be up-regulated in breast cancer cells lines and primary breast tumors and is considered a marker for increased tumor grade and nodal metastaisis [46]. Cells expressing Bcl-xL are more likely to survive following DNA damage and can potentially accumulate new somatic mutations at higher frequencies [47]. An increase in the Bcl-xL/ Bax ratio provides DNA damage induced resistance and survival to many cancer cell types [48]. Even though we observe an increase in the basal levels of mitochondrial depolarization and ROS generation in response to CSC vapor, these signals may be counteracted by increase in the Bcl-xL/ Bax ratio, which may increase the cellular survival of the cells due to selective pressure of the surrounding environment which enable them to escape apoptosis and acquire resistance to chemotherapeutic drugs.

Our current study does not rule out other mechanisms of resistance to cisplatin, such as decreased uptake, inactivation by nucleophilic compounds, or accelerated DNA repair, in CSC-exposed urothelial cells [49] . In addition, the precise mechanism of how AK3 sensitizes cells to cisplatin has not yet been clearly defined. Of relevance to the studies herein, the linkage of translational suppression of AK3 that mediate CSC vapor-induced toxicity and cisplatin resistance that has not been previously reported. However, new data links Pre-miR-181a and pre-miR-630 with modulation of both mitochondrial and post mitochondrial steps of the intrinsic pathway of apoptosis, including mitochondrial transmembrane potential dissipation, in cisplatin resistant lung cancer cells [50] . Studies are ongoing in our laboratory to understand these and other cellular pathways involved in the regulation of AK3 in response to CSC vapor and cisplatin.

## Materials and Methods

### Cell Culture

Normal uroepithelial cell line SV-HUC-1 (ATCC, Manassas, VA**)** and bladder cancer cell line SCaBER (ATCC, Manassas, VA**)** were maintained in F-12K medium and Dulbecco's modified Eagle's medium respectively (Invitrogen, Carlsbad, CA) supplemented with 10% fetal bovine serum (Clontech, Mountain View, CA). To study the vapor effect of the cigarette smoke condensate (CSC, Murty Pharmaceuticals, Inc., KY), cells were also grown in the smoking dedicated incubator without direct CSC treatment [21]. Cells that were grown in a normal incubator that did not have any cell lines treated with CSC are labeled as control or parental. Cells that were grown and passaged in the incubator in which cell lines were being exposed to CSC vapor are labeled as 6M treated cells or termed as passive smoked cells. The CSC (100%) was stored at −80°C. For every two CSC treated flask, one passive smoked flask was maintained. Hence forth the SV-HUC-1 and the SCaBER cells exposed to passive smoke were referred to as HUC1-6M and the SCaBER-6M respectively. AK3 expression plasmid was generated by cloning the ORF of the gene in the pcDNA3.1+/Hygro plasmid using *EcoRI* and *NotI* sites. All clones were sequenced to rule out any mutation.

### Cell Viability Assay

SV-HUC-1, SCaBER, HUC1-6M and SCaBER-6Mcells were grown in 35 mm, 6-well culture plates. The cells were transfected with AK3 expression plasmid or the empty control vector using Fugene HD (Roche, Indianapolis, IN) as per manufacturer's protocol. 24 h after transfection the cells were rinsed with Hanks' Balanced Salt Solution (HBSS) and treated with 0, 1,2,3,4 or 5 µg/ml cisplatin or vehicle control for 48 h in complete medium at 37°C in 5% CO_2_ incubator. After the desired time of exposure, the drug containing medium was aspirated, the cells were rinsed with HBSS and MTT assay was performed as described in [51]. Cell viability was also performed with Calcein AM (acetoxymethylester of Calcein, Invitrogen) assay with an excitation frequency of 480 nm and an emission frequency of 535 nm [52]. .All experiments were done in triplicate. All cells were released of passive smoking 24 h before each experiment unless otherwise mentioned.

### Colony formation assay

SCaBER and SCaBER-6M cells were transfected with either empty vector or AK3 expression vector in 6-well plates. 24 hrs later, cells were exposed to 100 µg/ml hygromycin to select positively transfected cells. Cells were cultured with hygromycin for 5 days. Cell colonies were then treated with 2 µg/ml cisplatin for 2 days and grew for additional 5 days. Colonies were fixed with 10% formalin and stained with 0.4% crystal blue.

### SiRNA Transfection

ON-TARGET*plus* SMARTpool control siRNA, AK3 siRNA were purchased from Dharmacon (Lafayette, CO) and cells were transfected with RNAiMAX reagent (Invitrogen, CA) according to the manufacturer's instructions. Briefly, cells were plated at a density of 20,000/well in 12-well plates. The following day, a stock solution containing 0.5 µl (20 µM) of siRNA (control, AK3) and 1 µl of transfection reagent per 250 µl of OptiMEM media (Gibco, Carlsbad, CA) was prepared and incubated at room temperature for 20 min. Media was aspirated from each well and replaced with 500 µl of antibiotic-free culture medium (DMEM/10% FBS) plus 250 µl of transfection mixture. Following an overnight incubation, the transfection mixture was replaced with complete culture media.

### Western Blot

Whole cell extracts of SV-HUC-1, HUC1-6M and HUC1-0.1%6M, SCaBER and SCaBER-6M and cells, were prepared using RIPA buffer (10 mM Tris pH 7.4, 150 mM NaCl, 5 mM EDTA, 1% Triton-X-100, 0.1% SDS) containing protease inhibitors (Roche, Indianapolis, IN cat # 1697498) and 1 mM PMSF. Western blot analysis was performed as described in [53] using 30 µg protein lysates for each protein antibody studied. Mitochondrial and cytoplasmic fractions were prepared as described in [54]. Nitrocellulose membranes were hybridized with primary antibodies and developed with Amersham developer as per the manufacturer's instructions.

### Measurement of Mitochondrial Membrane Potential Activity

We used a fluorescent dye JC-1 to measure changes in mitochondrial membrane potential [55]. Once loaded into the mitochondria, JC-1 undergoes aggregate formation in the regions of high potential. This method has been validated for a reliable analysis of Δ*Ψ*_m_ changes in mitochondria [56]. The green fluorescent JC-1 (5,5′, 6,6′-tetrachloro-1,1′,3,3′-tetraethylbenzimidazolylcabocyanine iodine, T-3168) exists as a monomer at low membrane potential. Mitochondrial function was monitored following treatment of SV-HUC-1, SCaBER, HUC1-6M and SCaBER-6M cells with cispaltin for 48 h. Briefly, after experiment treatment cells were incubated for 15 minutes at 37°C in the 5% CO_2_ incubator in the presence of 10 µM of the JC-1 and cells were analyzed using FACStar flow cytometer (Becton Dickinson, Franklin Lakes, NJ) as per manufacturer's instructions (Stratagene, La Jolla, CA).

### Determination of Reactive Oxygen Species

DCFH-DA ( Invitrogen, CA) was used for Reactive Oxygen Species (ROS) detection. DCFH-DA is cleaved intracellularly by nonspecific esterases to form DCFH, which is further oxidized by ROS to form the fluorescent compound DCF[57]. SV-HUC-1, SCaBER, HUC1-6M and SCaBER-6M cells were treated with or without cisplatin for 48 h, DCFH-DA working solution was added directly to the medium to reach 2 µM, and then incubated at 37°C for 30 min. Cells were then washed once, re-suspended in PBS and kept on ice for an immediate detection by FACScan (Becton Dickinson, USA). Mean fluorescence was generated using the software “Cell Quest” (Becton Dickinson, USA).

### Cytotoxicity assays

Membrane damage was evaluated by measuring the release of lactate dehydrogenase (LDH) in the culture medium [58]. SV-HUC-1, SCaBER, HUC1-6M and SCaBER-6M cells were treated with or without cisplatin for 48 h, before the media was collected for the LDH release assay. A control blank was run at all times without any cells. LDH is an oxidoreductase which catalyzes the inter-conversion of lactate and pyruvate. The assay is based on the reduction of NAD by the action of LDH. The resulting reduced NAD (NADH) is utilized in the stoichiometric conversion of a tetrazolium dye. The resulting colored compound was measured spectrophotometrically (A_490 nm_–A_690 nm_) using a Molecular Devices Spectra Max 250, 96 well plate reader (Sunnyvale, CA). The LDH-cytotoxicity detection kit (Roche Diagnostics, Indianapolis, IN) was used for all experiments. Each experiment was done in triplicate.

### Annexin V/PI staining

Apoptosis was determined by staining cells with annexin V-fluorescein isothiocyanate (FITC) and PI labeling as per manufacturer's instructions (Becton Dickinson, Franklin Lakes, NJ). Annexin V was used to detect early apoptotic cells during apoptotic progression. Briefly, SV-HUC-1, SCaBER, HUC1-6M and SCaBER-6M cells were treated with or without cisplatin for 48 h. The prepared cells were washed twice with cold PBS, re-suspended in 100 µ l of binding buffer at a concentration of 1×10^6^ cells/ml and stained with Annexin V and PI. The cells were analyzed on the FACStar flow cytometer (Becton Dickinson, Franklin Lakes, NJ). Data analysis was done using the software “Cell Quest”.

### Tumor Xenograft Study

Subcutaneous xenografts were generated in 5- to 6-week-old female athymic *nu/nu* mice (NIH) with SCaBER or SCaBER-6M (5×10^6^ cells/mouse) cells. One week following tumor cell inoculation, sixteen mice with successfully engrafted SCaBER or SCaBER-6M xenografts were randomized into two cohorts of eight animals each group and treated with either PBS or cisplatin (3 mg/Kg/dose, intraperitoneally). Tumor volumes were measured using digital calipers (Fisher Scientific) once per week and the mean tumor volume was calculated. Mouse experiments described here were done according to the guidelines of the Animal Care and Use Committee of Johns Hopkins Medical Institutions. Mice were maintained in accordance to the guidelines of the American Association of Laboratory Animal Care.
